# Influence of A-Site Deficiency and Ca Concentration on the Electrical and Crystallographic Properties of (Nd_0.2_Sr_0.7–*x*_Ca_*x*_)_*y*_Ti_0.95_Fe_0.05_O_3−δ_-Based Fuel Electrode for Solid Oxide Cells

**DOI:** 10.1021/acsaem.4c00824

**Published:** 2024-07-12

**Authors:** S. Paydar, K. Kooser, O. Volobujeva, S. Granroth, G. Nurk

**Affiliations:** † Institute of Chemistry, University of Tartu, Ravila 14a, Tartu 50411, Estonia; ‡ Institute of Physics, University of Tartu, W. Ostwaldi 1, 50411 Tartu, Estonia; § Department of Materials Science, Tallinn University of Technology, Ehitajate tee 5, 19086 Tallinn, Estonia; ∥ Department of Physics and Astronomy, University of Turku, 20014 Turku, Finland

**Keywords:** solid oxide fuel cell, A-site modified perovskite, conductivity, fuel electrode, MIEC

## Abstract

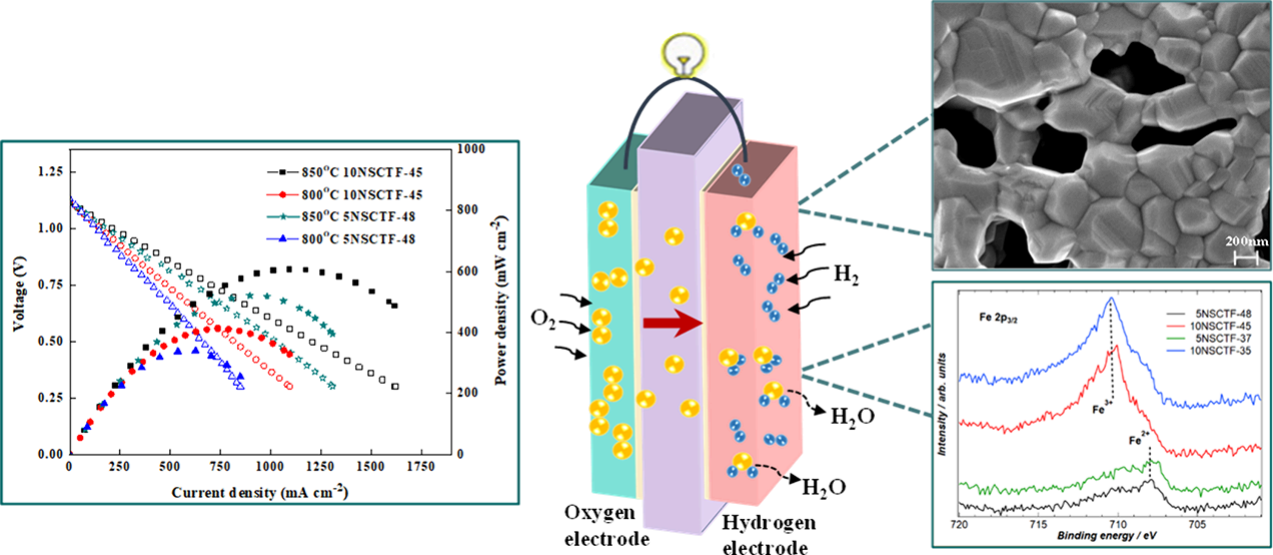

This study explores the impact of A-site deficiency and Sr/Ca ratio on the electrochemical and crystallographic properties of a (Nd_0.2_Sr_0.7–*x*_Ca_*x*_)_*y*_Ti_0.95_Fe_0.05_O_3−δ_ hydrogen electrode for solid oxide cells under reducing and air atmospheres. 5% and 10% A-site deficient (Nd_0.2_Sr_0.7–*x*_Ca_*x*_)_*y*_Ti_0.95_Fe_0.05_O_3−δ_ (*x* = 0.35–0.45, *y* = 1.05, 1) (referred to as 5NSCTF-*x* and 10NSCTF-*x*) materials were studied, while the ratio between A-site cations was kept the same with both deficiencies. The results demonstrate that the extent of A-site deficiency and the Ca concentration in the A-site have a significant impact on the microstructure (sinterability), conductivity, and catalytic activity of electrodes. Segregation of Nd from the lattice with 5% A-site deficiency was observed as a result of thermal treatment at low pO_2_. Among the studied materials, the highest total electrical conductivity of porous electrode layer at 850 °C and in 97% H_2_ + 3% H_2_O atmosphere was 4.8 S cm^–1^ observed for the Nd_0.2_Sr_0.35_Ca_0.35_Ti_0.95_Fe_0.05_O_3−δ_ (10NSCTF-35). The highest electrochemical performance was observed in the case of Nd_0.2_Sr_0.25_Ca_0.45_Ti_0.95_Fe_0.05_O_3−δ_ (10NSCTF-45), which showed a polarization resistance value equal to 0.19 Ω cm^2^ after 100 h of stabilization at 800 °C in a humidified (1.7% H_2_O) H_2_ atmosphere. The best electrochemical performance with 606 mW cm^–2^ power density at 850 °C in 98.3% H_2_ + 1.7% H_2_O atmosphere was demonstrated by a 50 wt % Nd_0.2_Sr_0.25_Ca_0.45_Ti_0.95_Fe_0.05_O_3−δ_ + 50 wt % Ce_0.9_Gd_0.1_O_2−δ_ composite.

## Introduction

The implementation of renewable energy sources stands as one of the principal challenges faced by modern society. The solid oxide fuel cell (SOFC) is an important component of future energy systems, offering efficient and fuel-flexible capabilities.^1−5^

State-of-the-art commercial systems are using traditional nickel-based anodes characterized by high conductivity and excellent catalytic activity. However, they encounter significant challenges, such as a high sensitivity to carbon deposition and sulfur poisoning, particularly in the presence of natural hydrocarbon fuels at intermediate operating temperature range (600–800 °C).^6−8^ In addition, moderate redox stability and Ni coarsening of the Ni-based cermet remain as the other major drawbacks.^9,10^ Aforementioned issues and the resulting relatively high system cost hold back large-scale commercial deployment of SOFC systems. Consequently, interest is increasing in alternative materials that could replace Ni-cermet fuel electrodes and address the mentioned issues. Within this realm, perovskite-type complex oxides (ABO_3_) with mixed ionic–electronic conductivity (MIEC) have emerged as a promising group of materials.^11^ Perovskites offer several advantages over Ni-cermet, including a wider electrochemically active region, enhanced tolerance to carbon and sulfur, and good redox stability.^11−13^ The properties of ABO_3_ perovskites are influenced not only by the transition metal cations occupying octahedral B-sites sites but also by the characteristics of A-site cations, often utilized in conjunction with rare-earth and alkaline-earth metals.^11^ MIEC materials employed in SOFC anodes can encompass p-type conductors, like Sr-doped LaCrO_3,_ or n-type semiconducting perovskites, such as La-doped SrTiO_3_. In recent years, La-doped SrTiO_3_ has gained significant attention for its exceptional dimensional stability, high conductivity, and remarkable resistance to sulfur poisoning and carbon deposition.^14,15^

The optimization of dopant concentrations on the A-site (such as La, Nd, etc.) and the B-site (such as Fe, Ni, etc.) of SrTiO_3_-based compounds offers the potential to enhance catalytic activity, electrical conductivity, and chemical stability of these materials.^16,17^ It has been reported that among perovskite structures with lanthanoids (Ln), like La, Pr, Nd, Sm, and Gd as dopants in the A-site of Ln_*x*_Sr_1–*x*_Co_*y*_Fe_1–*y*_O_3−δ_, the composition with Nd demonstrates the best electrical conductivity and very good catalytic activity for oxygen reduction.^18^ Additionally, Tamimi et al. observed that materials incorporating Nd exhibited lower electrode impedance compared to those with La in Ln_0.5_Sr_0.5_Co_0.8_Fe_0.2_O_3−δ_ (Ln = La, Pr, Nd). This observation was linked to the high oxygen mobility in Ln_0.5_Sr_0.5_Co_0.8_Fe_0.2_O_3−δ_ with Nd.^19^

Only a few attempts to dope the A-site of SrTiO_3_ (STO) with Nd have been conducted so far. Pradhan et al. reported that in the La_0.1_Sr_0.8–*x*_Nd_*x*_TiO_3−δ_ system the solubility of Nd dopant is limited, i.e., doping with Nd at higher concentrations than *x* = 0.09 leads to the incorporation of Nd at Ti^4+^ site, causing an unprecedented increase in the dielectric constant.^20^

The doping of A-site of STO with Ca has been proposed to bring the conduction orbitals of titanium closer to each other by reducing unit cell volume and thereby improving electronic conductivity.^21−23^

Experimental evidence has shown that A-site deficiency can improve both electronic and ionic conductivity of Ln_*x*_Sr_1–*x*_TiO_3−δ_ compounds.^24,25^ As a result of creating A-site deficiency, the concentration of oxide ion vacancies and reducibility of Ti^4+^ to Ti^3+^ (leads to higher electronic conductivity) increases compared to nondeficient lattice.^23,26−28^ Furthermore, A-site deficiency has also been used for mitigating Sr segregation, which may be a significant factor contributing to the deactivation of perovskite oxide surfaces and the subsequent degradation of SOC electrode performance.^29,30^

This study investigates the impact of A-site modifications on the electrical and electrochemical performance of two types of hydrogen electrodes: 5% and 10% A-site deficient Nd_0.21_Sr_0.74–*x*_Ca_*x*_Ti_0.95_Fe_0.05_O_3−δ_ (*x* = 0.37–0.48) and Nd_0.2_Sr_0.7–*x*_Ca_*x*_Ti_0.95_Fe_0.05_O_3−δ_ (*x* = 0.35–0.45), respectively (referred to as 5NSCTF-*x* and 10NSCTF-*x*). The study compares the properties of 5NSCTF-*x* with 5% A-site deficiency to those of 10NSCTF-*x* with 10% A-site deficiency, with precisely the same Ln/Sr/Ca ratios and employing identical synthesis and testing techniques for both types of electrodes.

## Experimental Section

### Synthesis of Materials

Nd_0.21_Sr_0.74–*x*_Ca_*x*_Ti_0.95_Fe_0.05_O_3−δ_ (*x* = 0.37–0.48) and Nd_0.2_Sr_0.7–*x*_Ca_*x*_Ti_0.95_Fe_0.05_O_3−δ_ (*x* = 0.35, 0.45) ceramic powders were synthesized using the glycine–nitrate combustion method and denoted as 5NSCTF-*x* and 10NSCTF-*x*, respectively, with 5% and 10% A-site deficiencies. High-purity chemicals, including Nd(NO_3_)_3_·6H_2_O (99.9%, Alfa Aesar), Sr(NO_3_)_2_ (99.9%, Alfa Aesar), Ca(NO_3_)_2_·4H_2_O (99.9%, Alfa Aesar), Fe(NO_3_)_3_·9H_2_O (99.9%, Alfa Aesar), and C_6_H_22_N_2_O_8_Ti 50% solution in water (Sigma-Aldrich), along with glycine (99%, Sigma-Aldrich) as reducing agent, were employed. The nitrate to glycine mole ratio was kept at 1:0.7. The exact concentration of each metal cation in the precursor solutions was determined using thermogravimetric analysis (STA 449 F3 Jupiter, Netzsch). The powder obtained from the glycine–nitrate combustion process was then calcined at 1100 °C for 20 h to form the perovskite-structured NSCTF. The exact stoichiometry of the synthesized powders with 5% and 10% A-site deficiencies is provided in Table 1. The production of the screen-printing paste from the raw powder involved the use of specific additives (the details can be found in our previous study^28^). Information about sintering temperatures, heating rates, and dwell times of all prepared layers is provided in Table S1.

**Table 1:** Stoichiometries and Abbreviations of Studied Nd_0.21_Sr_0.74–*x*_Ca_*x*_Ti_0.95_Fe_0.05_O_3−δ_ and Nd_0.2_Sr_0.7–*x*_Ca_*x*_Ti_0.95_Fe_0.05_O_3−δ_ Powders

stoichiometry of interest	abbreviation
Nd_0.21_Sr_0.37_Ca_0.37_Ti_0.95_Fe_0.05_O_3−δ_	5NSCTF-37
Nd_0.21_Sr_0.26_Ca_0.48_Ti_0.95_Fe_0.05_O_3−δ_	5NSCTF-48
Nd_0.2_Sr_0.35_Ca_0.35_Ti_0.95_Fe_0.05_O_3−δ_	10NSCTF-35
Nd_0.2_Sr_0.25_Ca_0.45_Ti_0.95_Fe_0.05_O_3−δ_	10NSCTF-45

### Electrical and Electrochemical Measurements

The electrical conductivity of the 5NSCTF-*x* and 10NSCTF-*x* porous electrodes was measured using the standard four-probe method. Alumina plates with a thickness of 250 μm were used as support for studying porous electrode layers. The 5NSCTF-*x* and 10NSCTF-*x* electrodes were applied to the alumina substrates using screen printing and covering a geometric surface area of 1 cm^2^. The porous layers were subsequently sintered at 1250 °C for 5 h, and uniform porous layers with a thickness of approximately 13–18 μm, depending on sinterability, were obtained (Figure S1). Four platinum (Pt) electrodes were then printed onto the porous MIEC electrode at precise distances and underwent subsequent thermal treatment.

The details of the fuel cell tests and symmetrical cell experiments can be found in the Supporting Information and in our previous study.^28^ All measurements were conducted using a Carbolite VST 1200 furnace over a temperature range from 650 to 850 °C. Electrochemical and conductivity measurements were performed using a Solartron 1287A potentiostat/galvanostat and a Solartron 1260 frequency response analyzer. Impedance spectra were recorded with an AC voltage amplitude of 10 mV and a frequency range spanning from 0.01 Hz to 100 kHz.

All electrochemical and conductivity measurements were conducted under gas overflow conditions to ensure consistent gas composition above the studied electrode throughout the experiments. Gas flow rates were regulated using EL-FLOW SELECT F-201CV mass flow controllers (Bronkhorst). During the electrical conductivity experiments and electrochemical measurements of symmetrical cells, two distinct gas compositions were employed: 1% H_2_ + 1.7% H_2_O + 97.3% Ar and 98.3% H_2_ + 1.7% H_2_O, which correspond to *p*O_2_ = 1.26 × 10^–17^ atm and *p*O_2_ = 1.3 × 10^–21^ atm at 850 °C and *p*O_2_ = 1.3 × 10^–22^ atm and *p*O_2_ = 1.34 × 10^–26^ atm at 650 °C, respectively. Gas mixtures of H_2_ and Ar were passed through water, and by changing the ratio between hydrogen and water, different gas compositions with varying partial pressures of oxygen were obtained.

Impedance analysis of fuel cells was conducted under precise fixed cell potentials (ranging from open circuit voltage to −0.9 V). Cyclic voltammograms were recorded at a scan rate of 5 mV s^–1^. Humidified hydrogen served as the fuel in the fuel electrode compartment. The fuel mixture was passed through a gas washing bottle containing Milli-Q+ water, which was maintained at 15 °C in a water circulator. This resulted in water uptake of 1.7%. During fuel cell measurements, the oxygen electrode compartment was supplied with synthetic air comprising 79% N_2_ and 21% O_2_.

### Physical Characterization of the Material

A Bruker-AXS D8 X-ray diffractometer was used for analyzing the crystal structure and phase purity. The X-ray diffractometer was equipped with a Cu Kα radiation source operating at 40 kV and 40 mA. Additionally, it included a Goebel mirror, 2.5° Soller slits, and a LynxEye 1D detector. Topas 6 software was used to determine the lattice parameters. XRD peaks were fitted using the Fundamental Parameters peak type, and known Au lattice parameters were used as a reference for comparison. The crystallite size of all pellets was calculated using the Scherrer equation.

To investigate the microstructural changes and elemental/chemical composition in the electrode during sintering and cell operation, high-resolution scanning electron microscopy (HR-SEM) and energy-dispersive X-ray spectroscopy (EDS) were employed. The HR-SEM analysis was conducted using the Zeiss Merlin system.

X-ray photoelectron spectroscopy (XPS) measurements for all samples were performed by the Thermo Scientific Nexsa system at Turku University. The Nexsa system utilizes a monochromatized Al Kα radiation beam and is equipped with a hemispherical energy analyzer. Curve-fitting analysis of all photoelectron spectra was carried out using the SPANCF package.^32,33^

## Results and Discussion

### Physical Characterization

**Figure 1:**
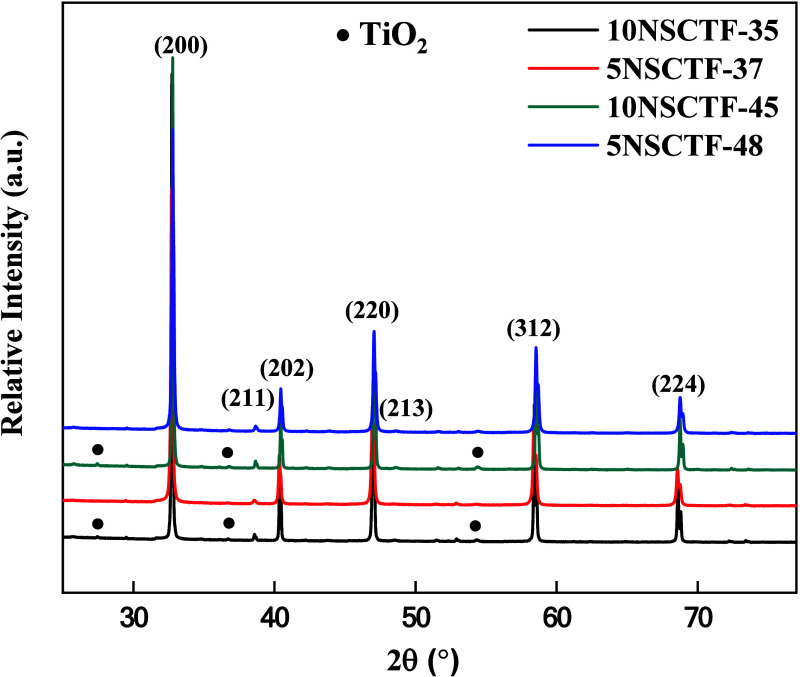
XRD pattern of Nd_0.21_Sr_0.74–*x*_Ca_*x*_Ti_0.95_Fe_0.05_O_3−δ_ (*x* = 0.37–0.48) and Nd_0.2_Sr_0.7–*x*_Ca_*x*_Ti_0.95_Fe_0.05_O_3−δ_ (*x* = 0.35–0.45) powders in the air at room temperature.

The XRD patterns (Figure 1) confirm the orthorhombic perovskite structure for all synthesized materials. The XRD patterns exhibited similar characteristics across all compositions. TiO_2_ as a secondary phase was observed in the 10NSCFT-*x* compositions sintered in air with very low-intensity reflections. To understand the influence of modifications in the A-site of Nd_0.21_Sr_0.74–*x*_Ca_*x*_Ti_0.95_Fe_0.05_O_3−δ_ (*x* = 0.37 and 0.48) and Nd_0.2_Sr_0.7–*x*_Ca_*x*_Ti_0.95_Fe_0.05_O_3−δ_ (*x* = 0.35 and 0.45), the unit cell volumes of all samples after calcination in air were calculated (Table 2). On the fitted XRD diffractograms (typical fit goodness values: *R*_wp_ = 3.8, GOF = 2.75), the Rietveld refinement technique was applied to assign the lattice parameters. As expected, the unit cell volume decreases when Ca concentration is increased (in both 5NSCTF-*x* and 10NSCFT-*x* compositions), attributed to the larger ionic radius of Sr^2+^ (1.32 Å)^34^ compared to Ca^2+^ (1.14 Å).^35^ An increase in A-site deficiency from 5% to 10% also results in a slight decrease in unit cell volume, as demonstrated also in previous studies.^30,36^

**Table 2:** Unit Cell Volume Size (Å^3^) of 5NSCTF-*x* and 10NSCTF-*x* after 20 h Calcination in Air at 1100 °C and after 20 h Calcination in Air at 1100 °C and Heat Treatment for 100 h at 1000 °C in a 100% H_2_ Atmosphere

composition	heat treatment atmosphere	5NSCTF-37	5NSCTF-48	10NSCTF-35	10NSCTF-45
volume size (Å^3^)	air	231.315	229.639	231.201	229.620
volume size (Å^3^)	100% H_2_	232.73	230.97	232.12	230.35

To explore the impact of reducing atmosphere on the crystallographic properties of studied materials, all powders were heat-treated in pure H_2_ at 1000 °C and analyzed using the XRD method. Diffraction patterns confirm the orthorhombic perovskite structure of studied samples, and in the case of 10NSCFT-*x*, samples show a small amount of Fe and Ti phase (Figure S3) after treatment in H_2_ at 1000 °C for 100 h. The calculated unit cell volumes of all reduced samples (Table 2) show an increase in unit cell volume due to the reduction of Ti^4+^ and the formation of oxide ion vacancies.

The morphology of the synthesized powders was evaluated by SEM (Figure S4). The raw powders were polydisperse and agglomerated. Shapes of particles indicate high crystallinity (Figure S4a–d). Based on the information from SEM micrographs, it is evident that 10NSCT particles are slightly bigger than 5NSCTF particles.

**Figure 2:**
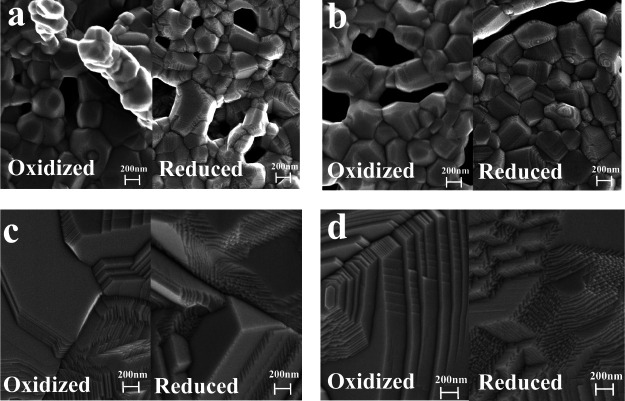
SEM images of porous (a) Nd_0.21_Sr_0.37_Ca_0.37_Ti_0.95_Fe_0.05_O_3−δ_ (5NSCTF-37), (b) Nd_0.21_Sr_0.26_Ca_0.48_Ti_0.95_Fe_0.05_O_3−δ_ (5NSCTF-48), (c) Nd_0.2_Sr_0.35_Ca_0.35_Ti_0.95_Fe_0.05_O_3−δ_ (10LSCTF-35), and (d) Nd_0.2_Sr_0.25_Ca_0.45_Ti_0.95_Fe_0.05_O_3−δ_ (10LSCTF-45) electrodes after 5 h sintering in air at 1250 °C and heat-treated for 100 h at 1000 °C in a 100% H_2_ atmosphere.

After sintering at 1250 °C for 5 h in the air (Figure 2), SEM micrographs were recorded from the 5NSCTF-*x* and 10NSCTF-*x* pellet surfaces. The SEM results demonstrate that an increase in A-site deficiency enhances significantly the sinterability of the material. Higher crystallinity and larger grain size are observed for 10NSCTF-*x* compared to 5NSCTF-*x*. Higher A-site deficiency facilitates ion diffusion during the sintering process, leading to more increased grain growth.^30^ The suppressed sinterability of 5% A-site deficient samples could also be explained by the segregation of Nd (as demonstrated by XPS results) from the bulk and the formation of the Nd-rich phase (with lower sinterability) close to the surface of samples. In fact, when the surface layer of one component forms (in this case the Nd-rich layer), then it suppresses the mobility of other ions of perovskite, and because of that, the grain growth is suppressed. These findings are well in line with the study by Li et al., who demonstrated that an increase in La^3+^ concentration in La_*x*_Sr_1–*x*_TiO_3−δ_ leads to a significant decrease in sinterability.^37^ The substitution of Sr^2+^ with Ca^2+^ in NSCTF lattice has no noticeable effect on sinterability (Figure 2).

To get first impression about the influence of the Sr/Ca ratio and A-site deficiency on the stability of studied materials in reducing atmosphere, the surfaces of 5NSCTF-37, 5NSCTF-48, 10NSCTF-35, and 10NSCTF-45 pellets were characterized using HR-SEM after 100 h treatment at 1000 °C in a H_2_ environment. The temperature was elevated from 850 °C (normal operating temperature) to 1000 °C to accelerate and amplify ion mobility processes. The HR-SEM micrographs of treated samples exhibit crystalline surfaces without additional spots or obvious segregated phases. However, detailed investigation and comparison of air- and hydrogen-treated material surfaces show morphological changes in surface structure (Figure 2). These changes indicate recrystallization of the electrode material at 1000 °C in a hydrogen atmosphere. In addition, the EDS mapping of electrode surfaces was performed, and a very slight increase of concentration in some locations on Ti and Fe maps was observed (Figure S5). These observations are in good accordance with XRD results of the samples studied after reduction, where slight reflections of Ti and α-Fe were observed.

### Photoelectron Spectra of Synthesized NSCTF Samples

X-ray photoelectron spectroscopy measurements were conducted to analyze the chemical composition of the studied samples. The photoelectron spectra of Nd (3d, 4s), Sr 3d, Ca 2p, Ti 2p, Fe 2p, and O 1s photoelectron lines were recorded. In addition, the valence spectra and C 1s signal were detected for the calibration of binding energy.

**Figure 3:**
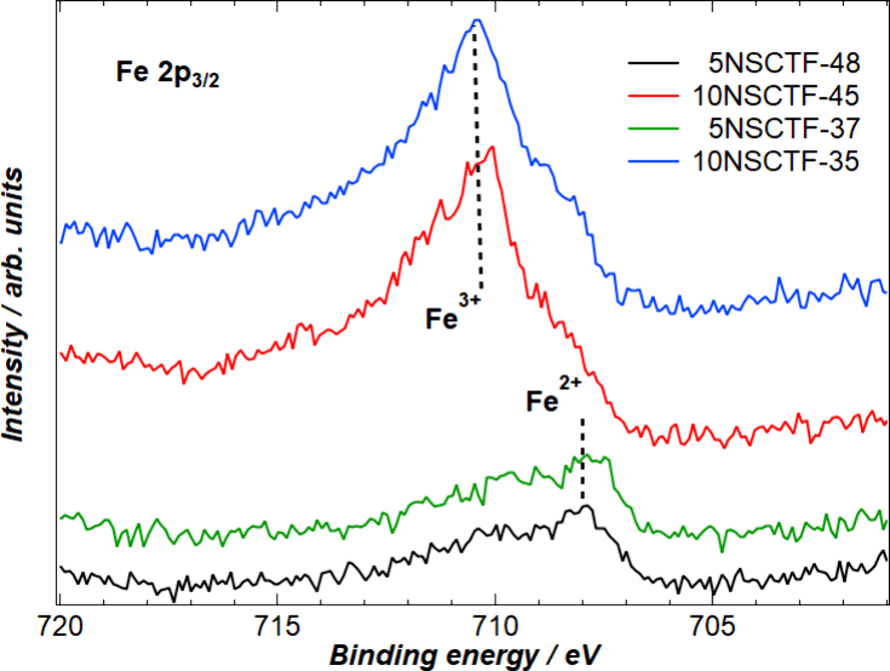
Photoelectron spectra of Fe 2p_3/2_ for all studied samples exposed to air after 5 h and at 1250 °C.

**Figure 4:**
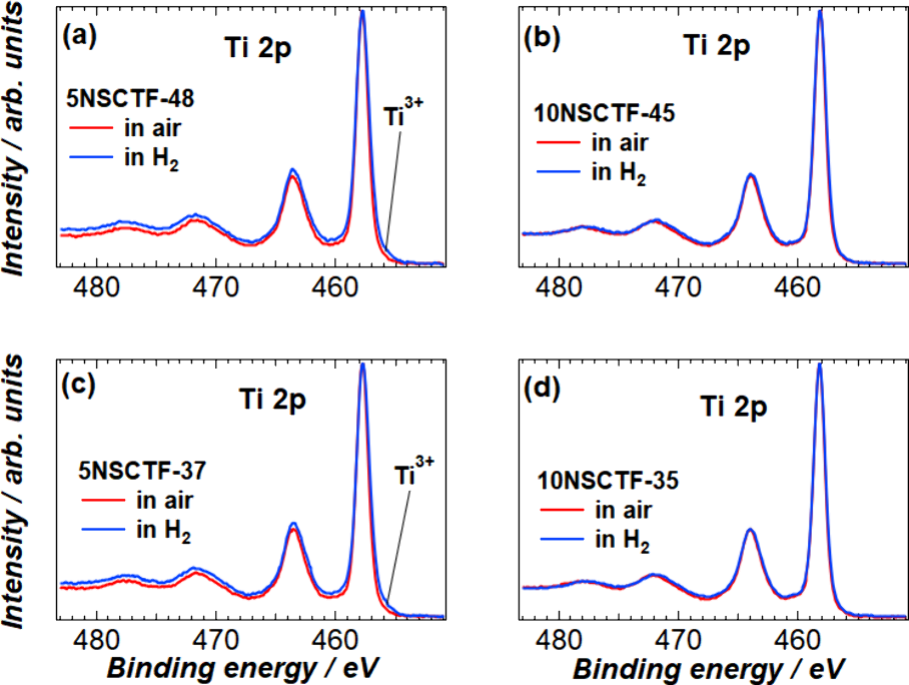
Ti 2p photoelectron spectra of (a) 5NSCTF-48, (b) 10NSCTF-45, (c) 5NSCTF-37, and (d) 10NSCTF-35 exposed to air and to H_2_ environments.

The most remarkable qualitative changes in photoelectron spectra were detected in the case of Fe 2p and Ti 2p (Figures 3 and 4). The atomic weight percentages of rare-earth metal Nd, alkaline-earth metals Sr and Ca, and transition-metal Ti at the surface of the studied samples were also estimated from the measured survey spectra. The concentration of Fe was so low that it is not included in Table 3. The atomic weight of the four main metallic elements (Nd, Sr, Ca, Ti) was estimated for both atmospheric conditions, for samples exposed in oxidizing (air) and in reducing (H_2_) environment (Table 3).

**Table 3:** Percentage of Atomic Weight of Nd, Sr, Ca, and Ti at the Sample Surface of the Studied Materials after Thermal Treatment in Reducing and Oxidizing Environments Compared to Expected Bulk Concentration (Calculated Atomic Weight %)

sample	photoline	atomic weight (%) exposed in air	calcd atomic weight (%)	atomic weight (%) exposed in H_2_
5NSCTF-48	Nd 4s	21.9	11.1	21.1
	Sr 3p	9.2	13.7	10.5
	Ca 2p	19.0	25.3	21.5
	Ti 2p	49.9	50.0	47.0
10-NSCTF-45	Nd 4s	12.9	10.8	9.6
	Sr 3p	12.5	13.5	9.1
	Ca 2p	22.5	24.3	18.3
	Ti 2p	52.1	51.4	63.1
5NSCTF-37	Nd 4s	23.3	11.1	22.5
	Sr 3p	12.8	19.5	13.8
	Ca 2p	14.8	19.5	16.6
	Ti 2p	49.2	50.0	47.1
10NSCTF-35	Nd 4s	12.4	10.8	13.5
	Sr 3p	17.1	18.9	13.9
	Ca 2p	17.7	18.9	7.3
	Ti 2p	52.8	51.4	65.4

The Fe 2p_3/2_ spectra of all four samples oxidized in air show quite drastic changes depending on the level of the A-site deficiency (Figure 3). First, the photoelectron signals of Fe 2p_3/2_ in a sequence of 5NSCTF-48, 10NSCTF-45, 5NSCTF-37, and 10NSCTF-35 have normalized intensities of 43%, 86%, 39%, and 100%, respectively. Thus, the iron signal is approximately 2–2.5 times weaker for the 5% A-site deficient electrodes compared to the 10% A-site deficient electrode materials.

The shape of Fe 2p_3/2_ spectra also indicates the different prevailing oxidation states of iron ions. The spectra of samples 5NSCTF-48 and 5NSCTF-37 have a pronounced maximum at the binding energy around 708 eV, but the other two, 10NSCTF-45 and 10NSCTF-35, have a much stronger maximum at 710 eV. According to ref (37) we can conclude that at the surface layer of the 5% deficient sample more Fe^2+^ ions and less Fe^3+^ ions appear, but at the surface of 10% A-site deficient electrodes, the Fe^3+^ concentration is higher compared to the Fe^2+^ concentration. The higher concentration of cations with higher oxidation state in materials with higher A-site deficiency can be attributed to the charge compensation effect, i.e., in material with higher A-site deficiency, the B-site elements are forced to change to higher oxidation state. The higher concentration of Fe ions at the surface of 10% deficient sample is apparent and well in line with studies about the influence A-site deficiency on the exsolution of B-site dopants.^38,39^

Figure 4 depicts the Ti 2p spectra normalized relative to the intensity of the Ti 2p_3/2_ peak. There are no remarkable changes in the spectral shape of the 10% deficient electrodes. However, the spectra of 5% deficient electrodes show a slightly increased full width at half-maximum of the Ti peaks due to the presence of the reducing (H_2_) atmosphere.

Broadening of Ti 2p peaks of 5% deficient materials as a result of thermal treatment at low *p*O_2_ (Figures 4a and 4c) is most likely caused by partial reduction of Ti^4+^ to Ti^3+^ and/or Fe^3+^ to Fe^2+^ and slight increase in lattice parameters because of this process. Moderate indications of Ti^3+^ electron structure in 455 eV binding energy region support this interpretation. In 10% deficient samples, no such changes (reduction of Ti^4+^ to Ti^3+^) occur in the surface layer as a result of thermal treatment in reducing atmosphere, the surface cations are dominantly in higher oxidation state and surface is stable. These results do not reflect accurately the situation at working conditions. However, it is clearly visible that higher A-site deficiency of bulk material forces the B-site cations at the surface to be more oxidized.

The atomic weight percentages of the main cations (Nd^3+^, Sr^2+^, Ca^2+^, Ti^3+/4+^) for each studied sample are given in Table 3. In 10% A-site deficient electrodes, the concentrations of material components in the surface of the material thermally treated in oxidizing (air) conditions are very similar to the expected bulk concentrations of the samples. The samples with 5% deficiency at A-site and thermally treated in air have approximately 2 times higher atomic contribution of Nd ions compared to expected concentrations. Segregation of Nd in materials with lower A-site deficiency is evident. Also, the Sr and Ca signals decreased by approximately one-fourth compared to the initial stoichiometric formulas of the 5% deficient materials. The amount of Ti signal in the 5% deficient electrodes is close to the initial stoichiometric values. These changes indicate that in 5% A-site deficient material, Sr and Ca are partially substituted by Nd in the top layer of the surface.

Thermal treatment in the reducing (H_2_) atmosphere causes remarkable changes in the concentrations of the main cations at the surfaces of all samples compared to the electrodes thermally treated in the air. On the surface of 5NSCTF-48 and 5NSCTF-37 samples, the signal of Ti is diminished slightly, i.e., by a few percentages, and there are moderate variations in the concentrations of the other elements, Sr, Ca, and Nd. In the 10% deficient electrodes, on the contrary, the atomic percentage of Ti is increased up to almost 63–65% compared to the initial stoichiometric value at around 51%. Besides the increase of Ti concentration, the amount of Sr, Ca, and Nd is decreased on the surfaces of 10% deficient electrodes. For the 10NSCTF-35 sample, the concentration of Ca is decreased more than twice, and the signal of Sr is lowered almost by one-fifth compared to the signal in oxidizing atmosphere.^40^

Considering the morphological as well as chemical changes of the surface because of thermal treatment in the reducing environment (Figure 2), it is very likely that electrode material with perovskite structure recrystallizes to different symmetry.

### Electrical Properties of Porous Electrode

To investigate the impact of A-site modifications on the electrical properties of fuel electrodes, the total electrical conductivities of porous 5NSCTF-*x* and 10NSCTF-*x* layers were measured over a temperature range of 650–850 °C at different oxygen partial pressures at two different gas atmospheres: 98.3% H_2_ + 1.7% H_2_O and in 1%H_2_ + 1.7% H_2_O + 97.3% Ar.

The conductivity values of porous 5NSCTF-*x* and 10NSCTF-*x* fuel electrodes were recorded after 24 h of *in situ* reduction in a hydrogen environment at 850 °C (Table 4). For all compositions of electrode materials, the electrical conductivity was thermally activated within the range of 650–850 °C in both 98.3% H_2_ + 1.7% H_2_O and in 1% H_2_ + 1.7% H_2_O + 97.3% Ar gas atmospheres (Figures S6a and S6b, respectively). These temperature-dependent behaviors prove the semiconductor-like characteristics of the studied materials.

**Table 4:** Electrical Conductivity (at 850 °C) and Activation Energies (650–850 °C) of 5NSCTF-*x* and 10NSCTF-*x* Samples in 98.3% H_2_ + 1.7% H_2_O and 1% H_2_ + 1.7% H_2_O + 97.3% Ar Atmospheres

composition	gas composition	5NSCTF-37	5NSCTF-48	10NSCTF-35	10NSCTF-45
conductivity (S cm^–1^)	98.3% H_2_ + 1.7% H_2_O	2.08	3.6	4.8	4.4
activation energy (eV)	98.3% H_2_ + 1.7% H_2_O	0.46	0.39	0.42	0.46
conductivity (S cm^–1^)	1% H_2_ + 1.7% H_2_O + 97.3% Ar	1.04	2.07	1.6	1.5
activation energy (eV)	1% H_2_ + 1.7% H_2_O + 97.3% Ar	0.6	0.53	0.57	0.64

Arrhenius plots were calculated for all 5NSCTF-*x* and 10NSCTF-*x* samples within the temperature range of 650–850 °C, and the corresponding activation energies were determined (Figure S6c,d and Table 4). The sufficient linearity observed in the Arrhenius plots for all samples indicates that the conduction mechanism remains relatively stable across the studied temperature range for each specific composition. The variation in activation energy (*E*_a_) of the conduction, influenced by material composition (A-site deficiency and Ca concentration) or gas atmosphere, reveals changes in the limiting conduction mechanism. The 10NSCTF-35 with 10% A-site deficiency displayed highest conductivity values, i.e., 4.8 S cm^–1^ in the 98.3% H_2_ + 1.7% H_2_O atmosphere at 850 °C (Table 4).

In general, the dependence of the total conductivity of the studied materials on the oxygen partial pressure and on A-site deficiency is similar to those demonstrated in previous studies of doped La_*x*_Sr_1–*x*_TiO_3−δ_: conductivity increases with the decrease of oxygen partial pressure and with an increase of A-site deficiency.^24,41^ Herewith, it should be mentioned that the effect of deficiency on conductivity is not influenced only through the defect chemistry but also through the microstructure of studied layer (which is significantly defined by A-site deficiency), i.e., more deficient layers are denser (bigger grains) because of better sinterability. Even if the total amount of material for layer cross section is the same, the 5% deficient layers with higher porosity have more and smaller grains, more grain boundary interfaces, and limiting bottlenecks.

As already mentioned above, the conductivity of the studied materials is significantly influenced by oxygen partial pressure. The alteration of the gas environment from a 98.3% H_2_ + 1.7% H_2_O gas mixture to a 1% H_2_ + 1.7% H_2_O + 97.3% Ar gas mixture results in a substantial 60–80% decrease in conductivity across the temperature range of 650–850 °C. The increase in electronic conductivity with a decrease of oxygen partial pressure, *p*O_2_, can be attributed to the creation of oxide ion vacancies resulting from oxygen release from the perovskite lattice accompanied by the reduction of Ti^4+^ to Ti^3+^, which contribute to the enhancement of the electronic conductivity of perovskite.^28,42^

The activation energies of conduction processes are smaller at lower oxygen partial pressures, indicating a higher proportion of electronic conductivity at these conditions.

The increase of A-site deficiency results in a higher concentration of oxide ion vacancies, leading to an increase in oxide ion conductivity as well as sinterability. However, high A-site deficiency might suppress the reduction of Ti^4+^ to Ti^3+^,^36^ which could also have a negative impact on electronic conductivity. This effect might explain the moderate total conductivity and relatively high activation energy (relatively high contribution of oxide ion conductivity) of 10NSCTF-35 and 10NSCTF-45 in a 1% H_2_ + 1.7% H_2_O + 97.3% Ar atmosphere. At very low *p*O_2_ (98.3% H_2_ + 1.7% H_2_O) and in the case of 10NSCTF-35 and 10NSCTF-45 samples, the reduction of Ti^4+^ to Ti^3+^ in bulk dominates over the effect caused by a deficiency.

In samples with 5% A-site deficiency, there is a positive effect of Ca dopant on electronic conductivity, i.e., higher Ca concentration leads to higher total conductivity and smaller activation energy. When the A-site deficiency is 10%, the influence of Ca doping is missing or is slightly the opposite.

### Electrochemical Analysis of Symmetric Cells

To assess the impact of A-site modification on the electrochemical properties of NSCTF-*x* electrodes and to investigate the initial midterm stability of these electrodes, impedance analysis was performed on symmetrical cells with 5NSCTF-*x* and 10NSCTF-*x* electrodes. The experiments were conducted under different oxygen partial pressures and temperatures, utilizing two distinct gas mixtures: 98.3% H_2_ + 1.7% H_2_O (corresponding to *p*O_2_ values of 1.3 × 10^–21^ atm at 850 °C and 1.34 × 10^–26^ atm at 650 °C) and 1% H_2_ + 1.7% H_2_O + 97.3% Ar (with *p*O_2_ values of 1.26 × 10^–17^ atm at 850 °C and 1.3 × 10^–22^ atm at 650 °C) in all sample measurements.

The two-electrode symmetric cell configuration was used, as described in the Experimental Section, for the comparison of polarization resistance (*R*_p_) values between 5NSCTF-*x* and 10NSCTF-*x* electrodes at nonpolarized conditions under constant *p*O_2_ and temperature conditions (Table 5). All *R*_p_ values were obtained from Nyquist plots (Figure S7) recorded at the end of a 100 h reduction/stabilization process at 800 °C (Table 5). The electrochemical studies of symmetric cells indicate a slight consistent increase in *R*_p_ values over time for all 5NSCTF-*x* and 10NSCTF-*x* electrodes exposed to the 98.3% H_2_ + 1.7% H_2_O gas mixture at 800 °C (Figure S7). The results of electrochemical monitoring during 100 h indicate that the stabilization process of most materials is not completed within this time frame.

**Table 5:** Polarization Resistance (*R*_p_), High-Frequency Resistance (*R*_HF_), and Low-Frequency Resistance (*R*_LF_) of 5NSCTF-*x* and 10NSCTF-*x* Samples in 98.3% H_2_ + 1.7% H_2_O Atmosphere after 100 h Stabilization at 800 °C and in 1% H_2_ + 1.7% H_2_O + 97.3% Ar Atmosphere and after 24 h Stabilization at 800 °C and Activation Energy Values in the Temperature Range from 650 to 800 °C

composition	gas composition	5NSCTF-37	5NSCTF-48	10NSCTF-35	10NSCTF-45
*R*_p_ (Ω·cm^2^)	98.3% H_2_ + 1.7% H_2_O	0.29	0.22	0.26	0.19
*R*_HF_ (Ω·cm^2^)	98.3% H_2_ + 1.7% H_2_O	0.03	0.04	0.04	0.04
*R*_LF_ (Ω·cm^2^)	98.3% H_2_ + 1.7% H_2_O	0.26	0.18	0.22	0.15
*E*_act_ (eV)	98.3% H_2_ + 1.7% H_2_O	0.79	0.79	0.85	0.85
*E*_actHF_ (eV)	98.3% H_2_ + 1.7% H_2_O	1.16	0.97	1.04	1
*E*_actLF_ (eV)	98.3% H_2_ + 1.7% H_2_O	0.62	0.63	0.69	0.67
*R*_p_ (Ω·cm^2^)	1% H_2_ + 1.7% H_2_O + 97.3% Ar	1.32	1.1	1.03	0.81
*R*_HF_ (Ω cm^2^)	1% H_2_ + 1.7% H_2_O + 97.3% Ar	0.03	0.03	0.04	0.04
*R*_LF_ (Ω·cm^2^)	1% H_2_ + 1.7% H_2_O + 97.3% Ar	1.29	1.07	0.99	0.77
*E*_act_ (eV)	1% H_2_ + 1.7% H_2_O + 97.3% Ar	0.46	0.44	0.53	0.49
*E*_actHF_ (eV)	1% H_2_ + 1.7% H_2_O + 97.3% Ar	1.23	1	1.26	1.16
*E*_actLF_ (eV)	1% H_2_ + 1.7% H_2_O + 97.3% Ar	0.33	0.32	0.36	0.39

10NSCTF-45 is the most active material (Table 5). It is apparent that the A-site modifications influence both the electrical conductivity of the material and the electrocatalytic properties of the surface.

The polarization resistance values at high frequency (HF) and low frequency (LF), *R*_HF_ and *R*_LF_, for the 5NSCTF-*x* and 10NSCTF-*x* samples were gained using the nonlinear least-squares fitting method (CNLS). The *R*_s_(*R*_HF_CPE_HF_)(*R*_LF_CPE_LF_) equivalent circuit was used for CNLS. The objective of this analysis was to clarify the influence of oxygen partial pressure, A-site deficiency, and Ca concentration on LF and HF limiting processes. The data in Table 5 represent the situation after a 100 h reduction process at 800 °C. To clarify how the changes in electrode composition and oxygen partial pressure on the electrode surface influence the mechanism of limiting processes, the activation energies were calculated from the Arrhenius plots of the LF and HF resistance values within a temperature range of 650–800 °C (Table 5).

According to the data presented in Table 5, the *E*_actHF_ (activation energy of the high-frequency process) values for 5NSCT-*x* and 10NSCT-*x* samples within the frequency range from 10^3^ to 10^2^ Hz are approximately in the range from 0.97 to 1.26 eV. This suggests that the HF process may be linked to various mass transport limitations of oxide ions occurring in the grain boundaries of the MIEC|electrolyte interface or within the MIEC|MIEC grains. Additionally, it may involve the diffusion of charged species to the triple phase boundaries (TPB) of the MIEC and electrolyte as well as the mass transfer of ions in the vicinity of the current collector.^27,43,44^ According to the literature, it is likely that high-frequency limiting process is gas–solid adsorption as a one step in surface-exchange phenomena.^28,45−47^ Activation energies of the low-frequency process within the frequency range from 100 to 0.01 Hz are around 0.63 eV at low *p*O_2_ and around 0.36 eV at high *p*O_2_. The variation in activation energy (*E*_actLF_) approves alterations in the structure of active adsorption sites due to changes in *p*O_2_.

As it can be seen from Table 5, the high-frequency resistance measured at 800 °C is slightly dependent on the A-site deficiency and increases with the increase of deficiency. These differences are very small and most likely related to the microstructural features of grain boundary region in the porous NSCTF electrode.

Ca concentration does not significantly influence the HF resistance value measured at 800 °C. However, the influence of Ca concentration on the activation energy is small but notable. A general trend observed is that increasing the Ca concentration from 35 to 45 wt % or 37 to 48 wt % results in a slight decrease in the activation energy of HF process. This phenomenon may be caused by the decrease of lattice parameters and the change of conductive properties of materials.

The LF resistance measured at 800 °C depends on A-site deficiency. Increasing of A-site deficiency from 5% to 10% leads to a decrease in LF resistance. The effect is smaller at low *p*O_2_ and higher at high *p*O_2_ conditions (Table 5). The activation energy of the LF process increases slightly with an increase of A-site deficiency. This phenomenon may be linked to the presence of Ti^3+^ ions in the electrode surface in the case of 5% A-site deficiency and lower thermal activation of LF process as a result of that but not necessarily; also, higher concentration of Nd is observable at 5% deficient electrodes.

Ca concentration has a significant influence on LF resistance measured at 800 °C. LF resistance decreased significantly with an increase of Ca concentration, whereas changes of LF activation energy with an increasing Ca concentration are only minor.

The influence of oxygen partial pressure on *R*_p_ (polarization resistance), *R*_HF_ (HF polarization resistance), and *R*_LF_ (LF polarization resistance) at *p*O_2_ = 1.1 × 10^–22^ atm and at *p*O_2_ = 1.1 × 10^–18^ atm (Table 5) was studied.

The *R*_HF_ of both 5NSCTF-*x* and 10NSCTF-*x* measured at 800 °C does not depend significantly on *p*O_2_. The activation energy of the HF process is only marginally influenced by *p*O_2_ and increases with oxygen partial pressure. These facts suggest that the HF process is likely linked to the oxide ion conductivity within the MIEC electrode.

LF resistance values (*R*_LF_) exhibit a significant increase with the rise in *p*O_2_, which could be caused by the higher oxide ion vacancy concentration at material surface at lower oxygen partial pressures or by higher concentration of reduced metal particles necessary for H_2_ adsorption.^21^ A gas mixture with low oxygen partial pressure contains a high concentration of hydrogen. The variation in activation energy (*E*_actLF_) also proves that the change of activity is not only caused by H_2_ concentration, but also the limiting mechanism or geometry of the active adsorption site is changed. These observations highlight the intricate interplay between oxygen partial pressure and the electrochemical processes within the studied materials. The study demonstrates the significance of A-site composition and deficiency as well as the gas environment in influencing the electrochemical behavior of the materials.

10NSCTF-45 and 5NSCTF-48, with high Ca concentrations, were identified as the most active materials, exhibiting the highest electrochemical properties under both gas compositions. Consequently, these materials were selected for testing in fuel-cell mode.

The symmetric cells were stabilized for 100 h at 800 °C and at *p*O_2_ = 1.1 × 10^–22^ before electrochemical characterization. After characterization, the monitoring of stability of these cells at higher oxygen partial pressure (*p*O_2_ = 1.1 × 10^–18^ atm) was carried out. The variation of *R*_p_ over time for all compositions is presented in Figure S8b. This experiment indicates that the electrochemical properties of the materials are relatively stable. The 5% deficient materials exhibit a slight change of *R*_p_ in time, but the *R*_p_ of 10% deficient material remains stable. A similar trend, i.e., an improvement on the stability by increasing the A-site deficiency, was observed by Li et al. if (Sr_0.3_La_0.7_)_1–*x*_(Fe_0.7_Ti_0.3_)_0.9_Ni_0.1_O_3−δ_ was studied.^48^

### Electrochemical Characterization of 5NSCTF-48 and 10NSCTF-45 Fuel Electrodes in Fuel Cell Mode

**Figure 5:**
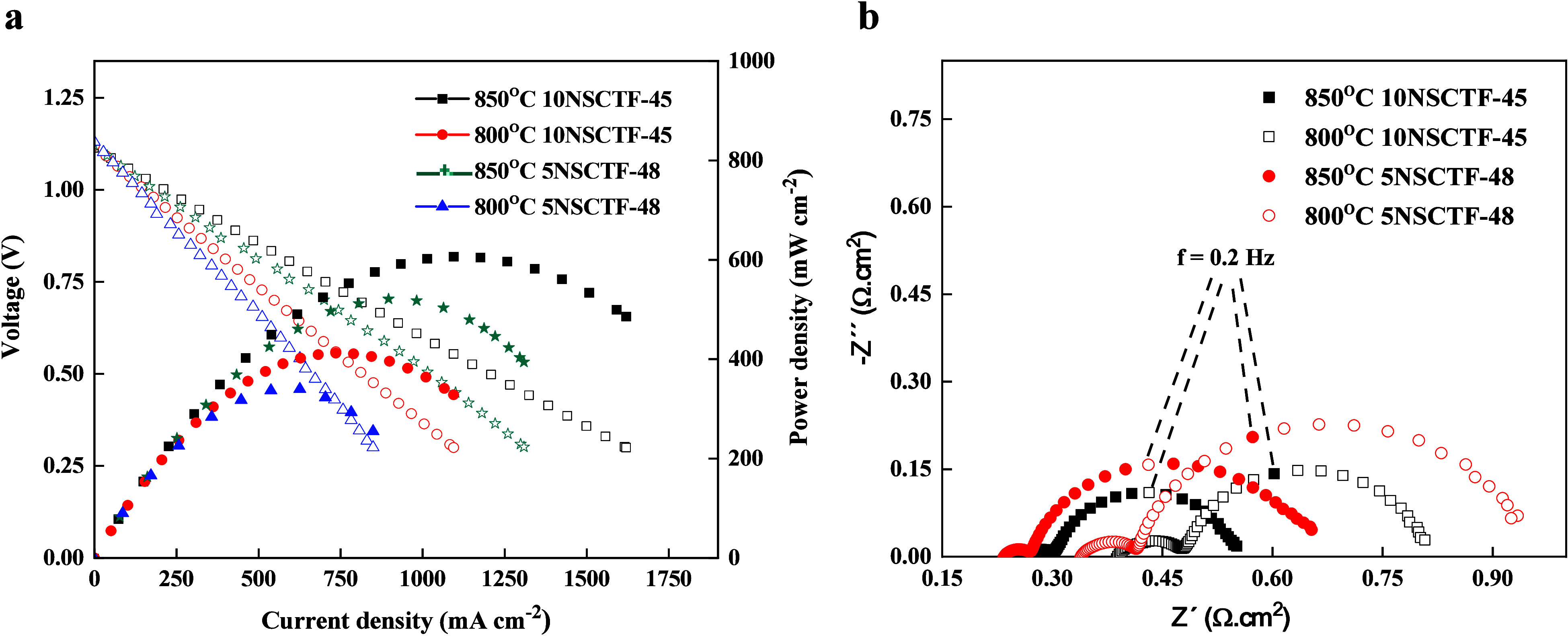
(a) *j*–*V* (scan rate 5 mV s^–1^) and *j*–*P* curves of fuel cell based on 5NSCTF-48 and 10NSCTF-45 at 850 °C and (b) Nyquist plots of fuel cells based on 5NSCTF-48 and 10NSCTF-45 measured in a 98.3% H_2_ + 1.7% H_2_O atmosphere at 850 and 800 °C.

To evaluate the electrochemical performance of the most active 5NSCTF-48 and 10NSCTF-45 anode materials under operando conditions, electrochemical solid oxide fuel cell (SOFC) tests were conducted using a 2-electrode configuration. Figure 5a illustrates the *j*–*V* (current–voltage) and *j*–*P* (current–power density) curves of two fuel cells: 5NSCTF-48 /(GDC)/GDC/ScCeSZ/GDC/LSCT and 10NSCTF-45 /(GDC)/GDC/ScCeSZ/GDC/LSCT, operating in a 98.3% H_2_ + 1.7% H_2_O atmosphere at 850 °C.

Only the initial fuel cell tests were done after initial 24 h stabilization, and the microstructure of the fuel electrode did not change during this test. Stabilization of such materials takes approximately 300–400 h, and understanding of stability of material needs several times longer experiments and separate projects.

The highest initial maximum power density (606 mW cm^–2^) is demonstrated in the case of fuel cell with the 10NSCTF-45 anode. The fuel cell with the 5NSCTF-48 anode exhibited a 14% lower initial power density of 521 mW cm^–2^ in comparison to 10NSCTF-45 (Figure 5a). The electrochemical impedance plot (Figure 5b) allowed polarization resistance (*R*_p_) to be determined. At 850 °C, the polarization resistance values for the cell with 5NSCTF-48 and 10NSCTF-45 were 0.43 and 0.29 Ω·cm^2^, respectively. At 800 °C, these values were 0.61 Ω·cm^2^ for 5NSCTF-48 and 0.42 Ω·cm^2^ for 10NSCTF-45. The smaller *R*_p_ values for 10NSCTF-45 align with the results obtained from the symmetrical cell tests (Table 5). Better *R*_s_ values for 5NSCTF-48 compared to the 10NSCTF-45 at moderate *p*O_2_ also align with the conductivity measurements.

## Conclusion

The impact of A-site modifications on the electrical properties, electrochemical performance, stability, and surface chemical composition of Nd_0.21_Sr_0.74–*x*_Ca_*x*_Ti_0.95_Fe_0.05_O_3−δ_ (*x* = 0.37–0.48) and Nd_0.2_Sr_0.7–*x*_Ca_*x*_Ti_0.95_Fe_0.05_O_3−δ_ (*x* = 0.35–0.45) fuel electrodes (referred to as 5NSCTF-*x* and 10NSCTF-*x*) in different gas atmospheres and feeing conditions were investigated. XRD results revealed that the unit cell volume of both 5NSCTF-*x* and 10NSCTF-*x* decreased with an increase in Ca concentration in the A-site. XPS analysis evidenced the segregation of Nd in materials with lower A-site deficiency under low *p*O_2_ condition. Four-probe conductivity measurements established the electrical properties of the porous fuel electrodes. The conductivity of 5NSCTF-*x* and 10NSCTF-*x* depended on the Ca concentration and A-site deficiency. Notably, the highest total electrical conductivity of 4.8 S cm^–1^ in a 98.3% H_2_ + 1.7% H_2_O atmosphere at 850 °C was achieved for 10NSCTF-35. The electrochemical performance of the synthesized ceramic materials was evaluated applying a two-electrode configuration. Electrochemical impedance spectroscopy measurements revealed that the composition exhibiting the highest catalytic activity was 10NSCTF-45, which displayed the lowest polarization resistance of 0.19 Ω cm^2^ after 100 h at 800 °C in a 98.3% H_2_–1.7% H_2_O atmosphere (*p*O_2_ = 1.1 × 10^–22^) under reducing conditions, with the lowest degradation during studied period. Under fuel cell working conditions, the 5NSCTF-48/(GDC)/GDC/ScCeSZ/GDC/LSCT and 10NSCTF-45/(GDC)/GDC/ScCeSZ/GDC/LSCT cells demonstrated high performance. The 10NSCTF-45 composition showed peak power densities of 606 mW cm^–2^ and an *R*_p_ value of 0.28 Ω·cm^2^ at 850 °C.

## Supporting Information Available

Additional experimental details, SEM images from symmetric cells, from used raw powders and from the samples used for the conductivity measurements, XRD pattern of Nd_0.2_Sr_0.7–*x*_Ca_*x*_Ti_0.95_Fe_0.05_O_3−δ_ powders after treatment in H_2_, EDS analysis results from surface of electrodes, electrical conductivity of materials and Arrhenius plots of porous 5NSCTF-*x* and 10NSCTF-*x* samples in reducing atmospheres, the *R*_p_ and Nyquist response of symmetrical cells exposed to reducing atmospheres (PDF)
