# Supplementary material for: Influence of A-Site Deficiency and Ca Concentration on the Electrical and Crystallographic Properties of (Nd0.2Sr0.7–xCax)yTi0.95Fe0.05O3−δ-Based Fuel Electrode for Solid Oxide Cells
Source: ACS Appl Energy Mater. 2024 Jul 12;7(14):5745–54. doi: 10.1021/acsaem.4c00824 (PMC11267496; doi:10.1021/acsaem.4c00824)
Supplement: Supplementary file 1 — ae4c00824_si_001.pdf [file ae4c00824_si_001.pdf]

Supporting information

Influence of A-Site Deficiency and Ca Concentration on the Electrical and Crystallographic Properties of $(Nd_{0.2}Sr_{0.7-x}Ca_x)_yTi_{0.95}Fe_{0.05}O_{3-\delta}$ Based Fuel Electrode for Solid Oxide Cells

S. Paydar[1], K. Kooser[2], O. Volobujeva[3], S. Granroth[4], G. Nurk[1*]

[1] *Institute of Chemistry, University of Tartu, Ravila 14a, Tartu 50411, Estonia*

[2] *Institute of Physics, University of Tartu, W. Ostwaldi 1, 50411 Tartu, Estonia*

[3] *Department of Materials Science, Tallinn University of Technology, Ehitajate tee 5, 19086 Tallinn, Estonia*

[4] *Department of Physics and Astronomy, University of Turku, 20014 Turku, Finland*

[*]E-mail addresses: gunnar.nurk@ut.ee

Experimental

Electrical and electrochemical measurements

The fuel cell tests, and symmetrical cell experiments were carried out on the $(Sc_2O_3)_{0.10}(CeO_2)_{0.01}(ZrO_2)_{0.89}$ (ScCeSZ) (Kerafol) electrolyte membranes with 250 μm thickness. A $Gd_{0.1}Ce_{0.9}O_{2-\delta}$ (GDC) (NexTech Materials) barrier layer was screen printed onto both sides of the electrolyte to avoid reactions between MIEC oxide material and zirconia. Fuel electrode with approximately 20 μm thickness and 0.50 cm^2 active area was screen printed onto GDC barrier layer of both sides for symmetrical cell tests. GDC (Fuel Cell Materials) powder (to improve ionic conductivity) mixed with 5NSCTF-x or 10NSCTF-x (1:1 wt% ratio) was used for preparing GDC-NSCTF composite electrodes. The screen-printing method was used to apply the raw pastes of electrode materials. The microstructure of one symmetrical cell is shown in Figure S2. For fuel cell measurements, $La_{0.6}Sr_{0.4}Co_{0.96}Ti_{0.04}O_{3-\delta}$ (LSCT) as a cathode was screen-printed symmetrically with an anode to the other side of the electrolyte membrane onto the GDC barrier layer. Complete description of cathode paste preparation has been presented in our earlier study [31]. In fuel cell setup platinum current collectors were screen printed onto both electrodes using commercial Pt-ink (Fuel Cell Materials). Solid oxide fuel cell tests were conducted for two-unit cells, 5NSCTF-48 and 10NSCTF-45 with the most active anode composition.

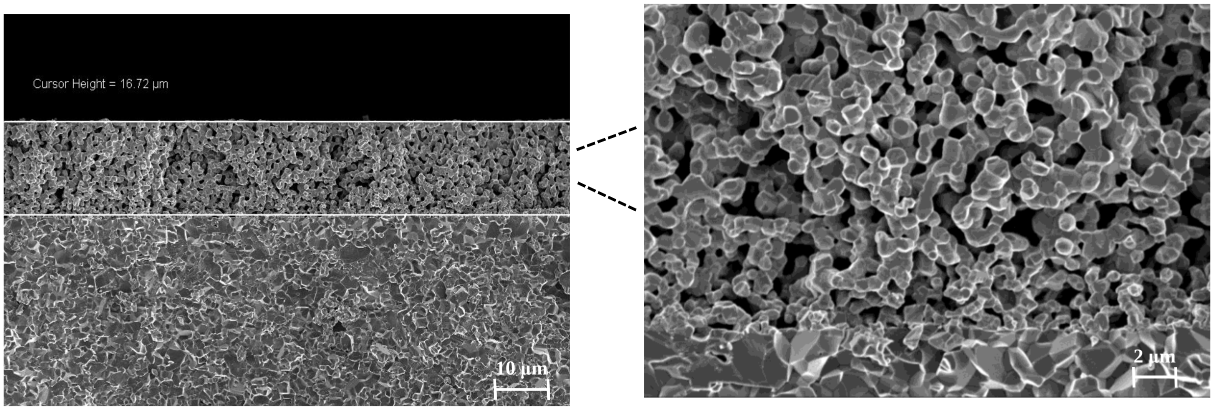

Figure S1. SEM images from the cross section of 5NSCTF-37 sample which were prepared for the conductivity test in two different magnifications.

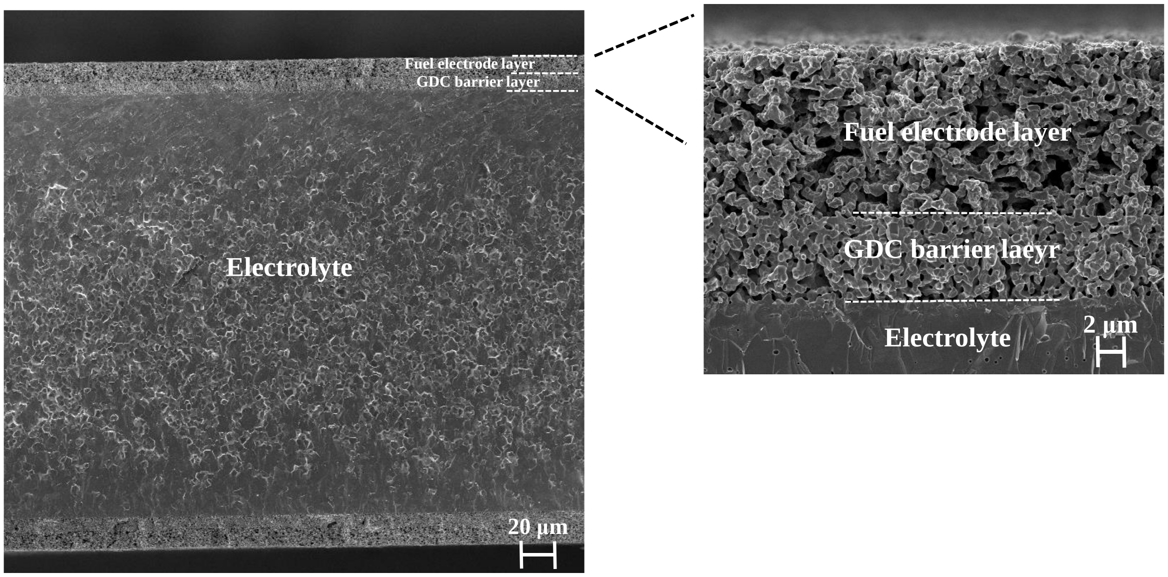

Figure S2. SEM images from the cross section of one symmetric cell with 5NSCTF-48 as electrode in two different magnifications.

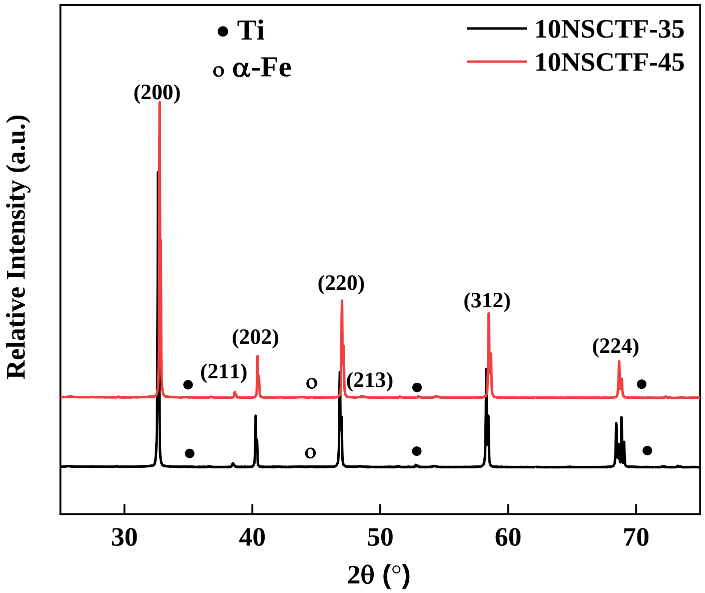

Figure S3. XRD pattern of $Nd_{0.2}Sr_{0.7-x}Ca_xTi_{0.95}Fe_{0.05}O_{3-\delta}$ powders after treatment in H_2 at 1000 °C during 100 h.

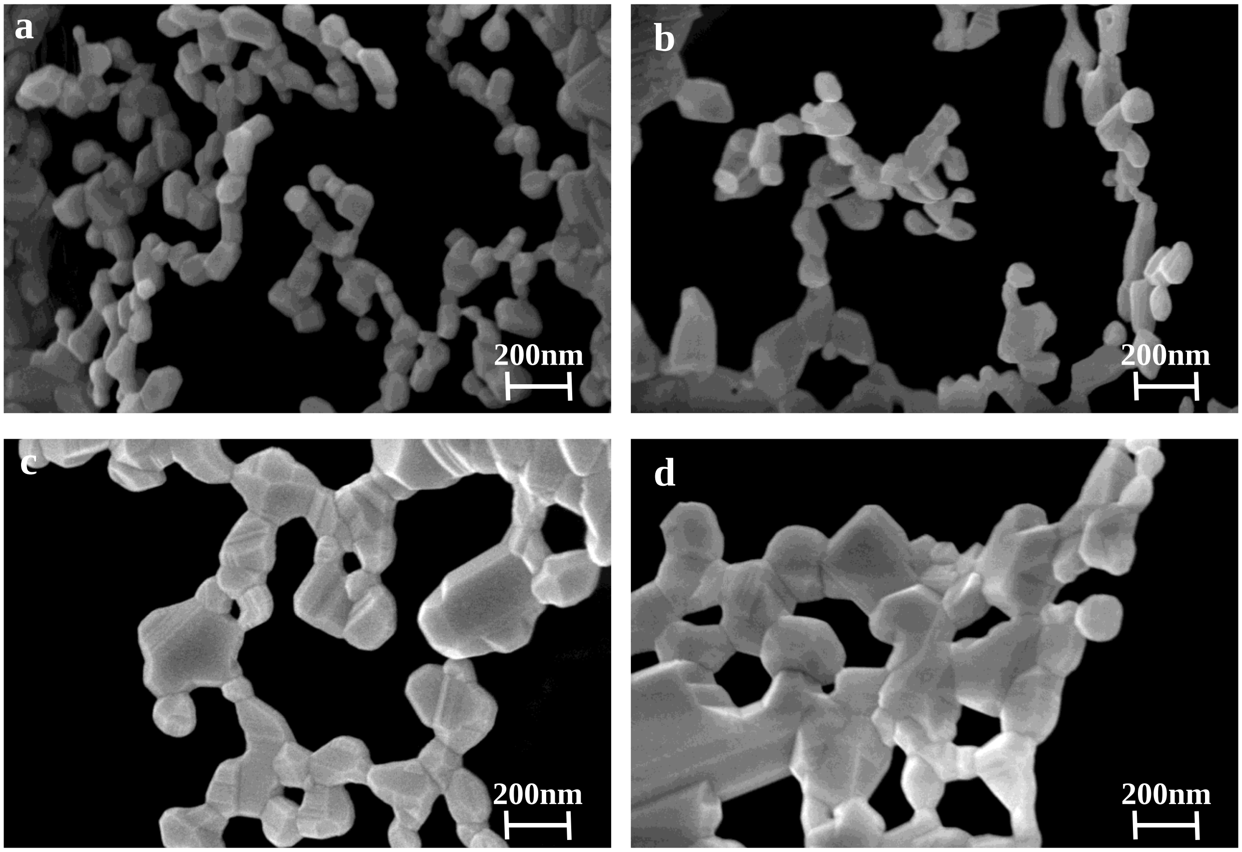

Figure S4. SEM images of synthesized powders (a) $Nd_{0.21}Sr_{0.37}Ca_{0.37}Ti_{0.95}Fe_{0.05}O_{3-\delta}$ (5NSCTF−37), (b) $Nd_{0.21}Sr_{0.26}Ca_{0.48}Ti_{0.95}Fe_{0.05}O_{3-\delta}$ (5NSCTF−48), (c) $Nd_{0.2}Sr_{0.35}Ca_{0.35}Ti_{0.95}Fe_{0.05}O_{3-\delta}$ (10LSCTF−35) and (d) $Nd_{0.2}Sr_{0.25}Ca_{0.45}Ti_{0.95}Fe_{0.05}O_{3-\delta}$ (10LSCTF−45).

(a)

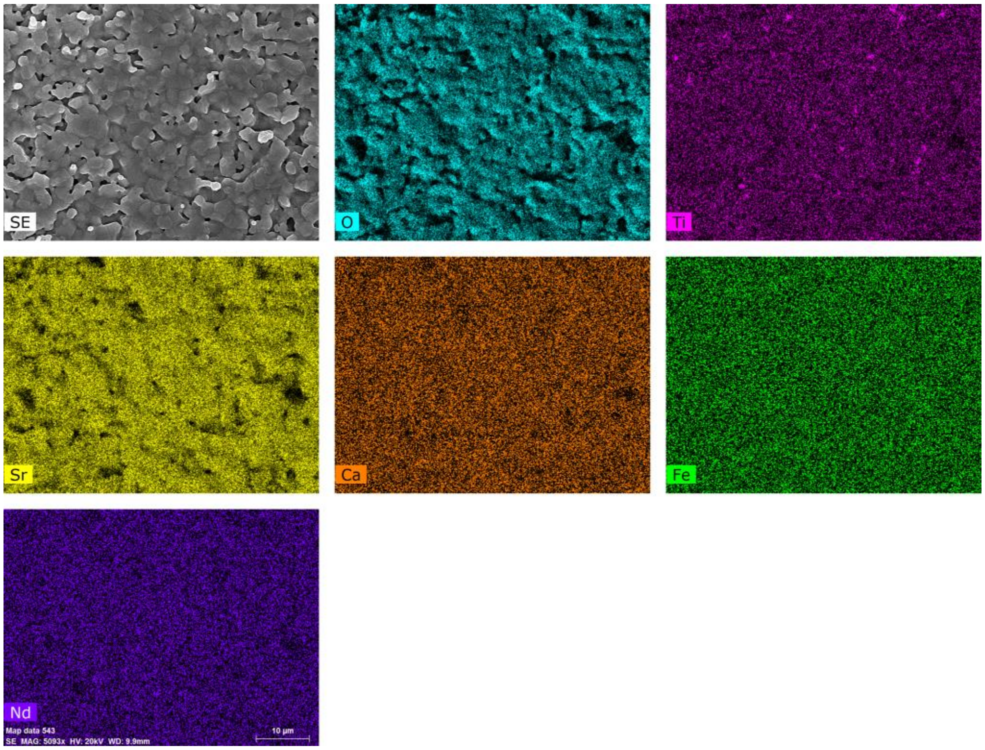
SE
O
Ti
Sr
Ca
Fe
Nd
Map data 543
SE MAG: 5093x HV: 20kV WD: 9.9mm
10 μm

(b)

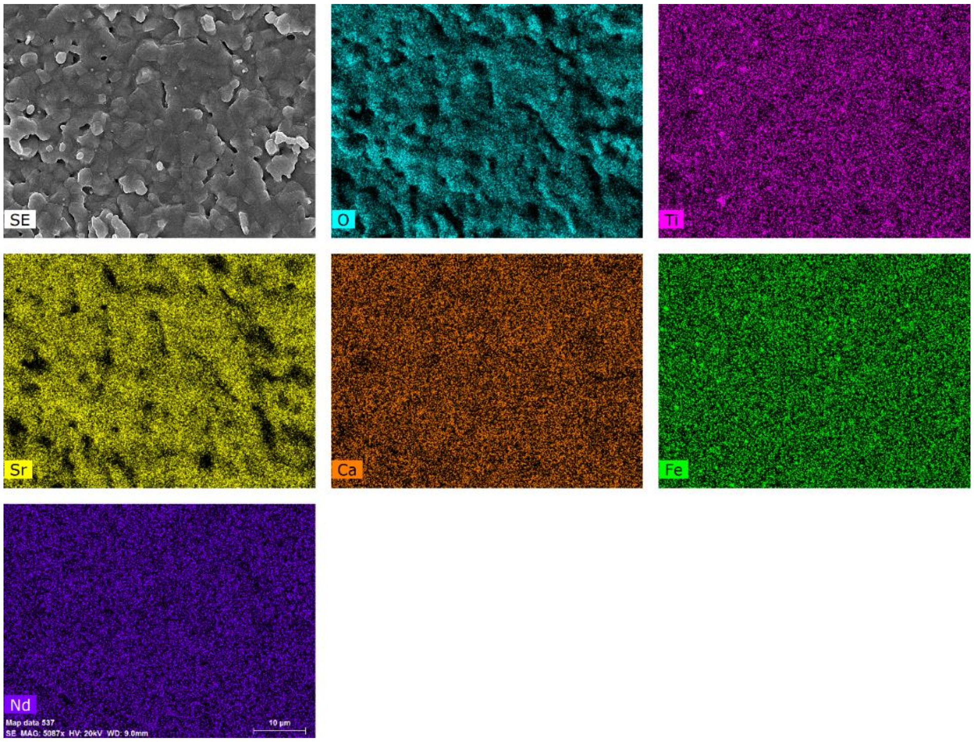
SE
O
Ti
Sr
Ca
Fe
Nd
Map data 537
SE MAG: 5087x HV: 20kV WD: 9.0mm
10 μm

Figure S5. EDS analysis of SEM images from surface of $Nd_{0.2}Sr_{0.25}Ca_{0.45}Ti_{0.95}Fe_{0.05}O_{3-\delta}$ (10LSCTF−45) electrodes (a) after 5h sintering in air and (b) after 5h sintering in air at 1250 °C and heat treated during 100 h at 1000 °C in 100% H_2 atmosphere.

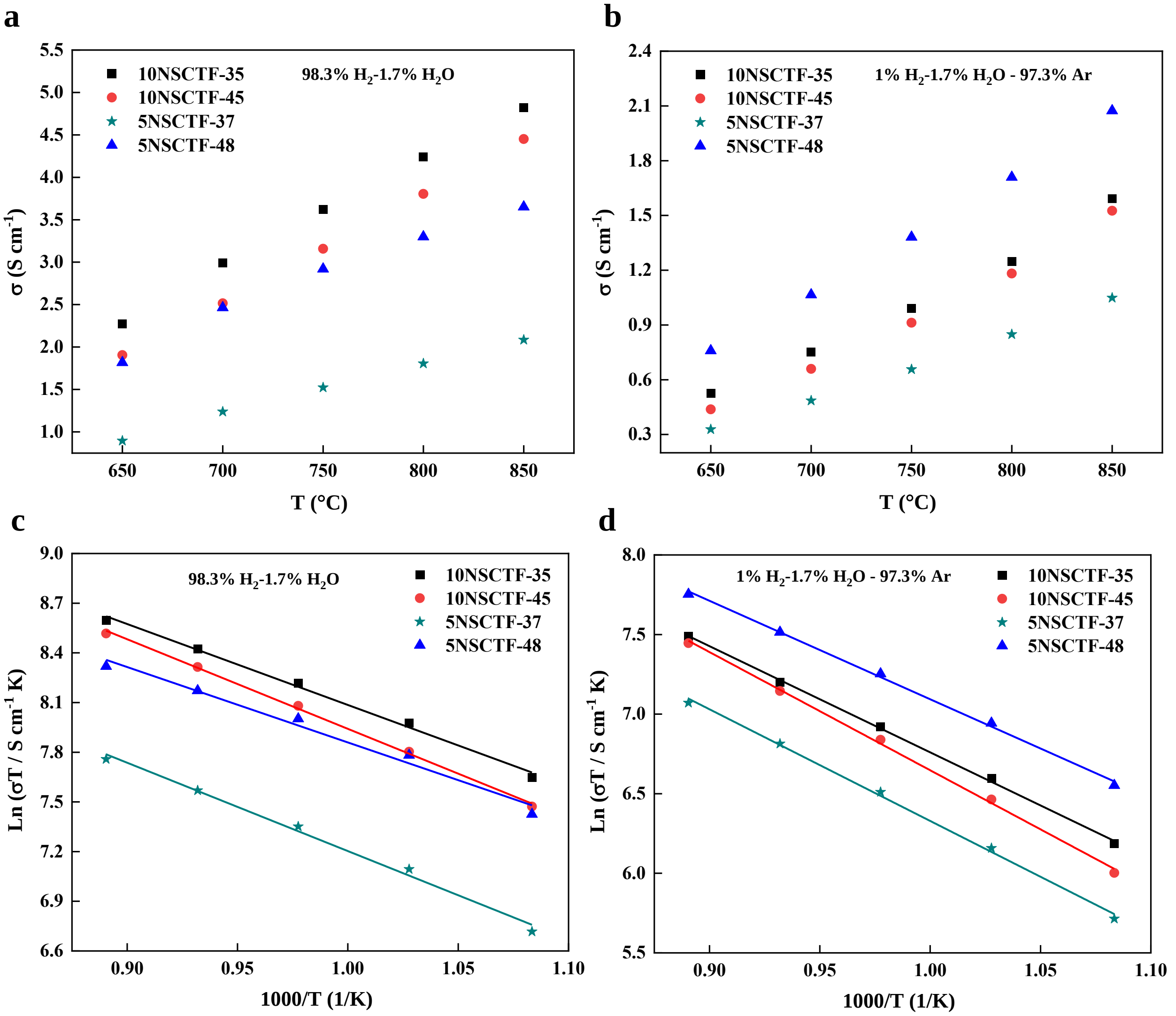

Figure S6. Electrical conductivity of 5NSCTF-x and 10NSCTF-x samples as a function of temperature in 98.3% H_2 + 1.7% H_2O (a) and 1%H_2 + 1.7% H_2O + 97.3% Ar atmospheres (b), and Arrhenius plots of porous 5NSCTF-x and 10NSCTF-x electrode conductivities in 98.3% H_2 + 1.7% H_2O (c) and in 1% H_2 + 1.7% H_2O + 97.3% Ar atmospheres (d).

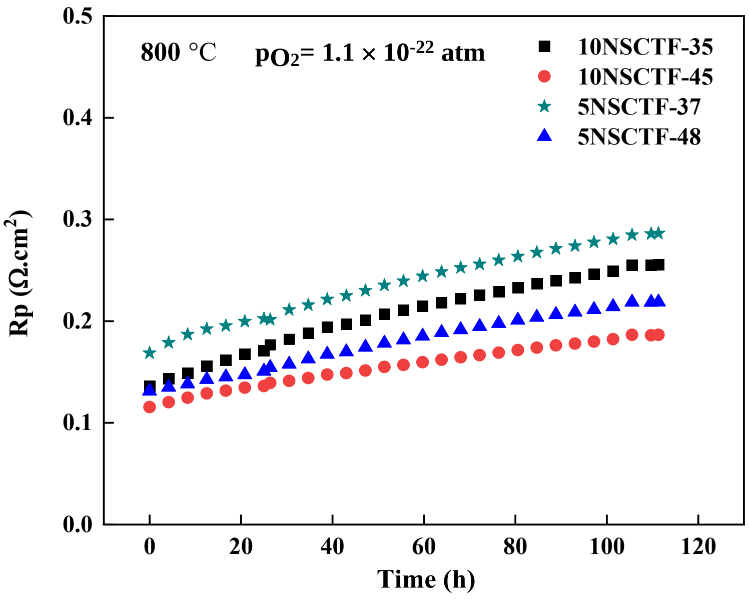

Figure S7. The R_p of symmetrical cell (5NSCTF-x (GDC)/GDC/ScCeSZ/GDC/5NSCTF-x (GDC)) and (10NSCTF5-x (GDC)/GDC/ScCeSZ/GDC/LSCTF5-x (GDC)) exposed to 98.3% H_2 + 1.7% H_2O (pO_2=1.1×10^{-22}) at 800 ℃ for 100 h.

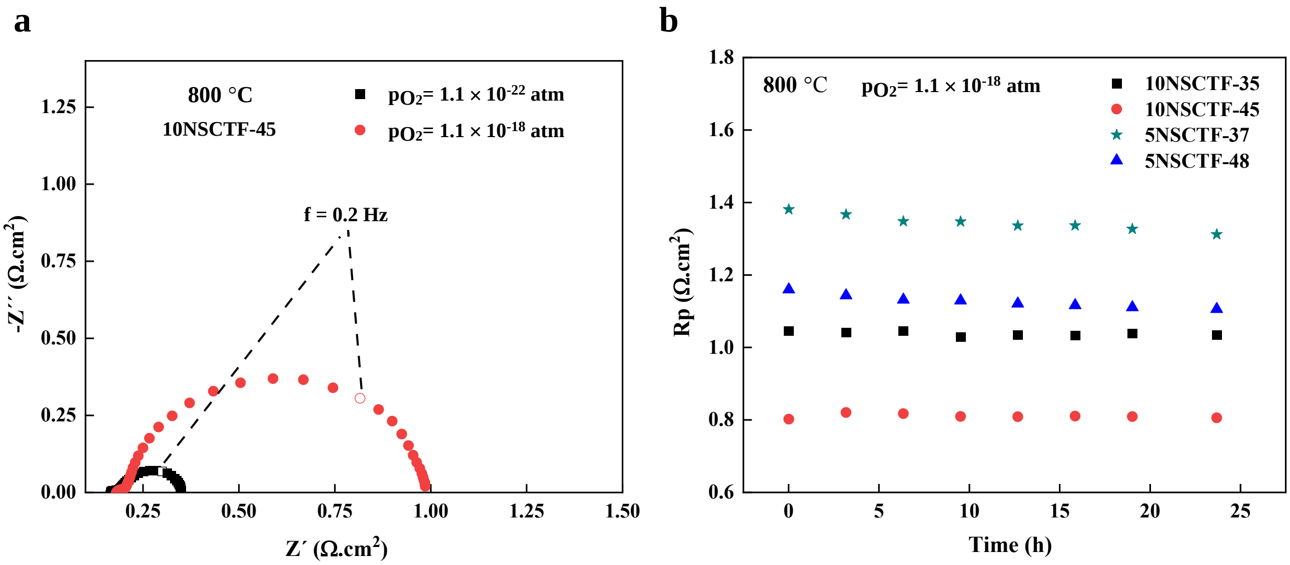

Figure S8. (a) Nyquist response of symmetrical cell (10NSCTF-45 (GDC)/GDC/ScCeSZ/GDC/10NLSCTF-45 (GDC)) exposed to 98.3% H_2 + 1.7% H_2O atmosphere (pO_2=1.1×10^{-22}) and 1% H_2 - 1.7% H_2O - 97.3% Ar (pO_2=1.1×10^{-18}) at 800 ℃, (b) the R_p of symmetrical cell (5NSCTF-x (GDC)/GDC/ScCeSZ/GDC/5NSCTF-x (GDC)) and (10NSCTF-x (GDC)/GDC/ScCeSZ/GDC/10NSCTF-x (GDC)) after stabilized for 100 h in 98.3% H_2 + 1.7% H_2O (pO_2=1.1×10^{-22}) atmosphere at 800 ℃, exposed to 1% H_2 + 1.7% H_2O + 97.3% Ar (pO_2=1.1×10^{-18}) at 800 ℃ for 24 h.

Table S1. Sintering temperatures, heating rate and dwell times for used materials.

**Material**	**Sintering temperature (°C)**	**Heating rate (°C /min)**	**Dwell time (h)**
$\mathbf{Gd_{0.2}Ce_{0.8}O_{2-d}}$	1300	2.5	5
$\mathbf{La_{0.6}Sr_{0.4}Co_{0.96}Ti_{0.04}O_{3-d}}$	1100	2.5	5
$\mathbf{Nd_{0.2}Sr_{0.7-x}Ca_xTi_{0.95}Fe_{0.05}O_{3-\delta}}$	1250	2.5	5
$\mathbf{Nd_{0.21}Sr_{0.74-x}Ca_xTi_{0.95}Fe_{0.05}O_{3-\delta}}$	1250	2.5	5
